# Enhanced Sensitivity and Detection of Near-Infrared Refractive Index Sensor with Plasmonic Multilayers

**DOI:** 10.3390/s21217056

**Published:** 2021-10-25

**Authors:** Tan Tai Nguyen, Nguyen Van Sau, Quang Minh Ngo, Gauthier Eppe, Ngoc Quyen Tran, Nguyen Thi Phuong Anh

**Affiliations:** 1 Department of Materials Science, School of Applied Chemistry, Tra Vinh University, Tra Vinh City 87000, Vietnam; 2 School of Basic Science, Tra Vinh University, Tra Vinh City 87000, Vietnam; nvsau@tvu.edu.vn; 3 Vietnam Academy of Science and Technology, University of Science and Technology of Hanoi, Hanoi 100000, Vietnam; ngo-quang.minh@usth.edu.vn; 4 Institute of Materials Science, Vietnam Academy of Science and Technology, Hanoi 100000, Vietnam; 5 Graduate University of Science and Technology, Vietnam Academy of Science and Technology, Hanoi 100000, Vietnam; 6 Mass Spectrometry Laboratory, MolSys RU, Department of Chemistry, University of Liège, Allée du Six Août, 11—Quartier Agora, 4000 Liège, Belgium; g.eppe@uliege.be; 7 Graduate University of Science and Technology, Vietnam Academy of Science and Technology, Ho Chi Minh City 700000, Vietnam; tnquyen979@gmail.com; 8 Institute of Applied Materials Science, Vietnam Academy of Science and Technology, Ho Chi Minh City 700000, Vietnam; 9 Faculty of Chemical Engineering, Ho Chi Minh City University of Technology, Ho Chi Minh City 700000, Vietnam; ntpanh369@gmail.com

**Keywords:** combination, multilayer, infrared laser, sensor, surface plasmon resonance

## Abstract

In this work, the multilayer of the surface plasmon resonance (SPR) sensor was optimized to achieve the maximum sensor sensitivity. By optimizing the thickness of the silver layer (Ag) and dielectric films (TiO_2_ and AlAs), the optimum sensitivity of the SPR sensor could be obtained. The performance of the SPR sensor proposed was compared with control simulations utilizing zinc oxide (ZnO) and molybdenum oxide (MoO_3_). The numerical results indicate that the figure-of-merits (FOM) of the SPR sensor was achieved around 150/RIU, corresponding to the sensor sensitivity of 162.79°/RIU with the optimized thicknesses of the TiO_2_, Ag, and AlAs layers of 140 nm, 60 nm, and 25 nm, respectively. This refractive index sensor shows the FOM to have high detection accuracy and high sensitivity that lead to finding potential application in bio-chemical detection with a small volume of liquid used in biological diagnosis.

## 1. Introduction

Over the last few decades, the optical detection technique has been widely used for biochemical applications for the study of biological binding and recognition [1,2,3,4]. Most studies have been focused on the development of the surface plasmon resonance (SPR) sensor. Typical SPR sensors have been implemented based on the Kretschmann configuration with a thin metal layer deposited on the prism [5,6,7,8,9] or optical fiber [10,11,12,13,14,15]. The change in the refractive index on the sensor surface can be indirectly recognized by measuring the wavelength or the angle at which the SPR condition is justified. In the reflected curve, the depth-to-width ratio and the change in wavelength or angle over the change in the refractive index demonstrate the figure–of–merits (FOM) of the SPR wave. Consequently, this SPR sensor structure is mainly applied for biomedical applications, i.e., the detection of fibrinogen for early disease diagnosis [16,17,18,19], glucose detection [20,21], foodborne bacterial pathogens detection [22,23,24,25], biotin–streptavidin binding [26], the detection of low–molecular–weight (<500 Da) biomolecules [27], and the detection of polypeptides [28]. It is noted that the SPR sensor with a multi-metal combination has shown a great benefit in terms of FOM. However, those configurations have relatively inherent drawbacks due to the strong energy of the visible light, which could damage the target biomolecules. To overcome this limitation, the use of infrared (IR) laser is a promising and attractive technique. Recently, the great benefit of SPR sensors utilizing IR laser has been reported [29,30,31,32].

The choice of metal layer for the surface is needed for optimizing the FOM of the sensor. Typically, gold (Au) and silver (Ag) are commonly used for SPR sensing platforms. The use of Au is widely adopted for the sensor surface due to its chemical stability [6,33,34]. However, the Au–coated SPR sensor is quite expensive. Alternatively, another promising material for SPR sensors is Ag, which has offered high sensitivity. However, Ag can easily oxidize [35,36]. In addition, dielectric materials (i.e., titanium dioxide (TiO_2_), molybdenum oxide (MoO_3_), and zinc oxide (ZnO)) have offered great benefits for the enhancement of sensor sensitivity by combining them with plasmonic materials [37,38,39]. Those materials have numerous advantages due to their smaller real permittivity magnitude than metals [37,38,39]. Recently, MoO_3_ has been utilized for a chemo-resistive sensor for the selective monitoring of H_2_ [38]. Moreover, portable optical sensors are also interesting for next-generation computing based on edge computing [40]. This leads to the investigation of a new SPR sensing platform based on a multilayer structure at a lower frequency range. 

This work aims to enhance the sensor’s FOM with a new SPR structure based on a prism with a three-layer combination, comprising TiO_2_/Ag/aluminum arsenide (AlAs)/sensing medium for foodborne bacterial detection. The theoretical modeling and analysis are also presented with a detailed description. The design structure is optimized based on different modulation thicknesses of Ag layer and dielectric materials (TiO_2_ and AlAs), with a refractive index of sensing in the medium range of 1.33–1.351 (RIU). The change in refractive index corresponds to an *E. coli* concentration of 10^3^ cfu/mL [41]. Furthermore, the performance of the SPR sensor was compared with the established SPR configurations (ZnO and MoO_3_). The results show that the SPR sensor performance with a wavelength of 1064 nm exhibited an enhancement of the FOM, i.e., sensitivity and detection accuracy for the combination structure of prism/TiO_2_/Ag/AlAs. 

## 2. Materials and Methods

**Figure 1:**
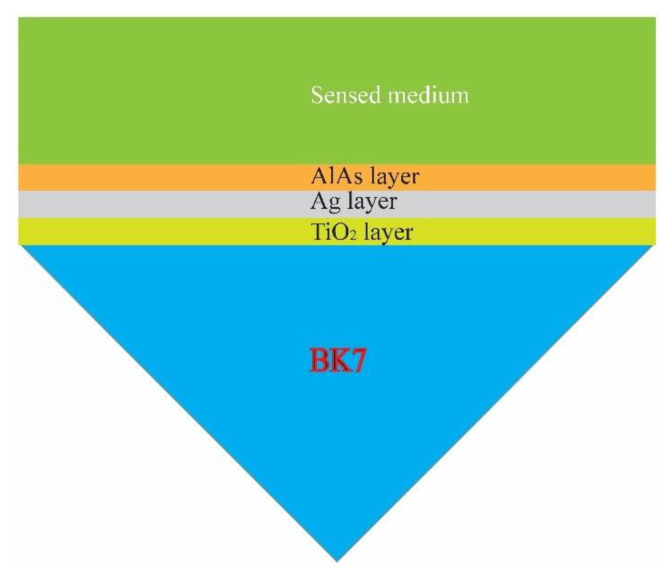
Sketch of the proposed sensor comprising the stacked layers of prism/TiO_2_/Ag/AlAs.

A BK7 prism was utilized for the design of the optical sensor, which is depicted in Figure 1. The multilayer structure proposed comprises prism/TiO_2_/Ag/AlAs with the semiconductor layer exposed to sensing mediums of varying refractive indices. The additional layer of TiO_2_ enhanced the adhesion between the plasmonic Ag layer and the prism and achieved high efficiency in light trapping for plasmonic excitation [37]. In addition, an enhanced SPR detection was achieved by incorporating an AlAs layer on top of a thin Ag film [42,43,44]. This is an effective alternative in bypassing the oxidation of an Ag layer [45]. It should be noted that those materials can be deposited on the prism (BK7) by using a thermal evaporation system or a magnetron sputtering technique [12,46]. 

For excitation of the surface plasmon wave, a collimated beam of IR laser with a wavelength of 1064 nm was incident to the prism surface by passing through a polarizer to obtain the transverse magnetic (TM) polarization light. To perform the simulation for the multilayer optical sensor, the simulated parameters are given in Table 1. 

The transfer matrix regression method was utilized to evaluate the reflection coefficient for the multilayer model of the sensor structure. In this model, the relationship between tangential field components at the first and the last interfaces is given below [52,53]

(1) $$\left[\begin{array}{c} E_{t1}\\ H_{t1} \end{array}\right]=M\left[\begin{array}{c} E_{t3}\\ H_{t3} \end{array}\right]$$

where *E_t_*_1_ and *H_t_*_1_ are the electric and magnetic field components at the first interface (TiO_2_/Ag). Similarly, *E_t_*_3_ and *H_t_*_3_ are the electric and magnetic field components at the third interface (AlAs/sensing medium). In addition, *M* is the characteristic matrix of the *N*-layer combination, as given below

(2) $$M=\prod_{k=2}^{N-1}M_{k}=\left[\begin{array}{cc} M_{11} & M_{12}\\ M_{21} & M_{22} \end{array}\right]$$

where

(3) $$M_{k}=\left[\begin{array}{cc} \cos\beta_{k} & \left(-i/q_{k}\right)\sin\beta_{k}\\ -iq_{k}\sin\beta_{k} & \cos\beta_{k} \end{array}\right]$$

(4) $$q_{k}=\frac{{\left(\varepsilon_{k}-\varepsilon_{BK7}\sin^{2}\varphi \right)}^{1/2}}{\varepsilon_{k}}$$

(5) $$\beta_{k}=\frac{2\pi d_{k}}{\lambda }{\left(\varepsilon_{k}-\varepsilon_{BK7}\sin^{2}\varphi \right)}^{1/2}$$

The reflection coefficient of TM field (p-polarization) and its amplitude are given below, respectively,

(6) $$r_{q}=\frac{\left(M_{11}+M_{12}q_{s}\right)+q_{BK7}-\left(M_{21}+M_{22}q_{s}\right)}{\left(M_{11}+M_{12}q_{s}\right)+q_{BK7}+\left(M_{21}+M_{22}q_{s}\right)}$$

(7) $$R={\left|r_{p}\right|}^{2}$$

where *k* is denoted for the *k*th layer; *d_k_* is the thickness of the *k*^th^ layer; *ε_BK7_* is the dielectric constant of the prism used; *ε_k_* is the dielectric constant of the *k*^th^–layer; φ is the incident angle of the laser light; λ is the wavelength of the laser; and *ε_s_* is the dielectric constant of the sensing medium. 

After the laser light was reflected from the sensor surface, the SPR characterization was investigated based on the reflectance versus the incident angle. The resonant angle corresponded to the minimum reflectance. The resonant angle and the minimum reflectance depended on the excitation wavelength, the thicknesses of the metal (Ag), semiconductor (AlAs), and dielectric (TiO_2_) layers, and the refractive index of the sensing medium (water). The thickness of all layers, including Ag, AlAs, and TiO_2_, were investigated for optimizing the sensor structure under the change in refractive index of the sensing medium. In this work, the sensor sensitivity was estimated based on the ratio between the change in the resonant angle (Δ*φ*) of the reflection curve and the change in the refractive index of the sensing medium (Δ*n*). From the simulated results concerning the reflectance as a function of incident angle, we estimated the sensor sensitivity, detection accuracy, and FOM as given below,

(8) $$S=\frac{\Delta \varphi }{\Delta n}$$

(9) $$DL=\frac{1}{FWHM}$$

(10) FOM=S×DL

where *S* is the sensor sensitivity and *n* is the refractive index of the sensing medium; *DL* is the detection accuracy and *FWHM* is the full-width at haft-maximum.

## 3. Results and Discussion

**Figure 2:**
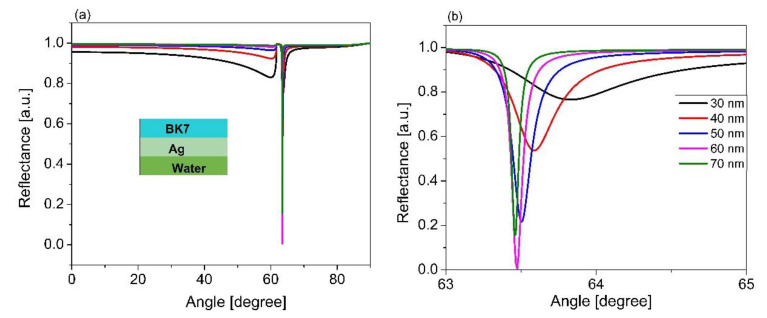
(**a**) Simulated results of Ag thickness from 30 nm to 70 nm; (**b**) magnified view of (**a**) from 63 to 65 degrees.

Most recent studies have focused on the optimization of the metal layer thickness, and it was demonstrated that the thickness of the metal layer strongly depends on the sensor structure. In the present study, the thickness of the Ag layer was optimized in the range from 30 to 70 nm with an increment of 10 nm, under the excitation of the IR laser with a wavelength of 1064 nm. Figure 2 presents the SPR characterization curve, considering that the BK7 prism was coated with a thin layer of Ag and covered by distilled water with a refractive index of 1.33 (RIU). In Figure 2b, it is observed that the resonant angle slightly shifted due to a change in the thickness of the metal layer. Moreover, the reflectivity of the SPR curve changes due to a change in the thickness of the Ag layer. The optimization thickness of the Ag layer was considered based on the reflectivity, with a minimum value or reflectance towards zero at the resonant angle at which most electromagnetic wave energy transfers to the surface plasmon wave. 

**Figure 3:**
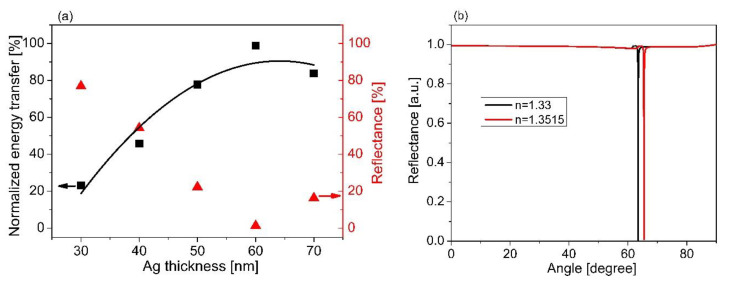
(**a**) The relationship between energy transfer and reflectance. (**b**) The change in resonance for different RI of the sensing medium.

Figure 3a shows the estimation of the reflectivity strength based on the SPR characteristic curve. It indicates that the optimum thickness of Ag is around 60 nm at the resonant angle of 63.5° (Figure 2b). It is worth mentioning that the sensor sensitivity is also one of the factors used for considering the optimization thickness of the Ag layer. In the present case, the refractive index of the sensing medium was set from 1.33–1.351 (RIU), corresponding to the change from DI water to an *E. coli* concentration of 10^3^ cfu/mL [38]. The sensitivity of 92.1°/RIU was obtained for the sensor structure comprising prism/Ag (Figure 3b). Note that the sensor sensitivity was estimated based on the change in the resonant angle over the change in the refractive index of the sensing medium.

To find an ideal characteristic shape for energy transfer based on the thickness of the Ag layer’s change, we used the parabolic curve with a quadratic second−order equation *E_T_* = *αt_Ag_*^2^ + *βt_Ag_* − *E_o_* to fit the simulated data in Figure 3a. The fitting results show that the minimum energy transfer (*E_o_*) was estimated at around 163.22 (a.u.), as shown in Table 2. The optimized thickness of Ag was obtained at 60 nm thickness with the resonant angle of 63.4° (Figure 3b), corresponding to a maximum energy transfer of 799.78 (a.u.). Moreover, the correlation coefficients (*R*^2^) were higher than 0.85, indicating that the model proposed, i.e., the second−order quadratic model, fit well into the simulated data.

**Figure 4:**
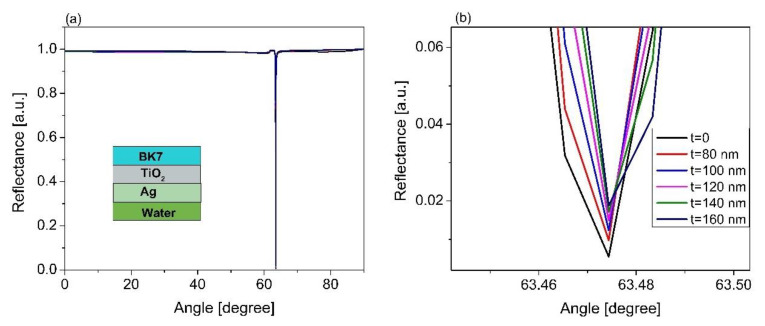
(**a**) Simulated results of TiO_2_ thickness changed from 80 nm to 160 nm based on the sensor structure of the BK7/TiO_2_/Ag/sensing medium; (**b**) magnified view of (**a**).

The TiO_2_ layer was introduced to the sensor structure between the Ag layer and the BK7 substrate, with the optimum thickness of Ag (60 nm). Figure 4a shows the corresponding SPR characteristic curve based on the sensor structure of the BK7/TiO_2_/Ag/sensing medium for different thicknesses of TiO_2_ from 80 to 160 nm. It was observed that the SPR resonant angle did not change, and the contrast of the SPR curve was lightly changed (Figure 4b). The sensor properties were estimated and are shown in Table 3. The results show that the sensitivity did not depend on the thickness of the TiO_2_ layer. However, the sensor detection accuracy was associated with the TiO_2_ thickness. When the thickness of TiO_2_ increases, the accuracy of the sensor detection is increased. The maximum detection accuracy of 15.87/°, which was 1.14 times higher than that of the other cases using various thicknesses of TiO_2_, i.e., thicknesses of 80, 100, and 120 nm, was obtained based on the TiO_2_ thickness of 140 nm. The TiO_2_ layer not only enhances the sensor detection accuracy, but it also increases the adhesion between the Ag layer and prism [37].

**Figure 5:**
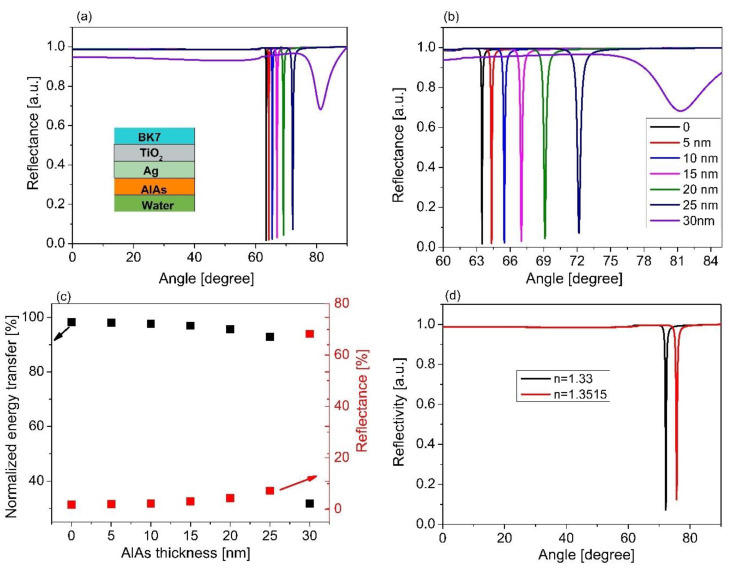
SPR spectra for varying thicknesses of AlAs based on the sensor structure of the BK7/TiO_2_/Ag/AlAs/sensing medium; (**b**) magnified view of (**a**); (**c**) the relationship between energy transfer and the AlAs thickness; (**d**) the change in resonance for different RI of the sensing medium based on the sensor structure of BK7/TiO_2_ (140 nm)/Ag (60 nm)/AlAs (25 nm).

**Figure 6:**
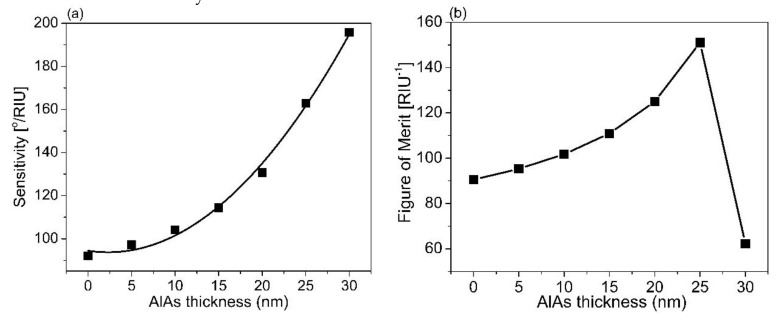
(**a**) The dependence of sensor sensitivity versus the thickness of the AlAs layer; (**b**) The comparison of the FOM of the sensor performance for various AlAs thicknesses.

Based on the results obtained, the Ag thickness of 60 nm and the TiO_2_ of 140 nm were used to estimate the thickness of the AlAs layer. We scanned the AlAs thickness from 5 to 30 nm to maximize FOM for a given thickness of Ag and TiO_2_, as mentioned above. Figure 5 displays the reflectance for varying the AlAs thickness. When the AlAs thickness was increased, the SPR angle slightly shifted and the reflectance was slightly decreased. Based on those results, the sensor sensitivity and the FOM were estimated, as seen in Figure 6. The results show that an increase in the AlAs thickness was associated with an increment in the sensor sensitivity (Figure 6a).

The parabolic shape with a quadratic second-order equation *S* = *S_o_* + *ax* + *bx*^2^ was fitted to the estimated results in Figure 6a to find the ideal characteristic parabolic relationship of the sensor sensitivity with the AlAs thickness during the sensor operating process. The fitting results show that the minimum possible sensitivity (*S_o_*) based on the multilayer structure was around 94.45°/RIU for the case of the AlAs layer, as illustrated in Table 4. The results also show that the optimum sensor sensitivity of 162.79°/RIU in the case of the AlAs layer (thickness of 25 nm) increased 43.42% in comparison without utilizing the AlAs layer. In addition, this sensitivity was also better than that of the other cases using various thicknesses of AlAs layer, i.e., thicknesses of 5, 10, 15, and 20 nm with the sensitivity increments of 40.29%, 36.0%, and 19.71%, respectively. Moreover, the correlation coefficient (*R*^2^) was higher than 0.98, indicating that the model proposed, i.e., the second-order quadratic model, fit well into the estimated data. 

Based on this prediction model, the thicker the AlAs layer is, the more sensitive the sensing performance is. However, when an increment in the AlAs layer was over 25 nm, this caused an increment in reflectance of around 70%. The reflectivity was higher than 50%, leading to the fact that there was a smaller coupling effect between the TM wave and the evanescent wave to generate SPR. This led us to obtain a lower value of FOM, as shown in Figure 6b. The maximum FOM was obtained around 150/RIU with the AlAs layer of 25 nm thickness. It is worth mentioning that this value of FOM obtained with an AlAs thickness of 25 nm was 41.06% and 60.26% higher than that of the case without using the AlAs layer and the AlAs layer of 30 nm thickness, respectively, as illustrated in Figure 6b. It is generally seen that the larger the FOM is, the better the sensitivity is. This result obtained is higher than that of other works using a plasmonic gold layer. Table 5 represents a comparison of the sensor proposed with the existing plasmonic sensors, which were found in the literature. In this case, we made a comparison of several aspects, i.e., the sensor structure, RI range, FOM, and operating wavelength. It can be generally seen that the sensor proposed shows better FOM than the existing sensors [54,55,56,57,58,59,60,61,62,63,64,65], which used expensive materials such as Au-coated fiber for SPR excitation. This form of a multilayer (TiO_2_ (140 nm)/Ag (60 nm)/AlAs (25 nm)) can be suitable for the implementation of a SPR sensor based on BK7 glass subtrate for an improvement in the detection efficiency of a small amount of bio-chemical agents, due to the enhancement of the evanescent field’s penetration depth into the sensing medium, leading to an enhancement of the sensor sensitivity and the FOM. 

**Figure 7:**
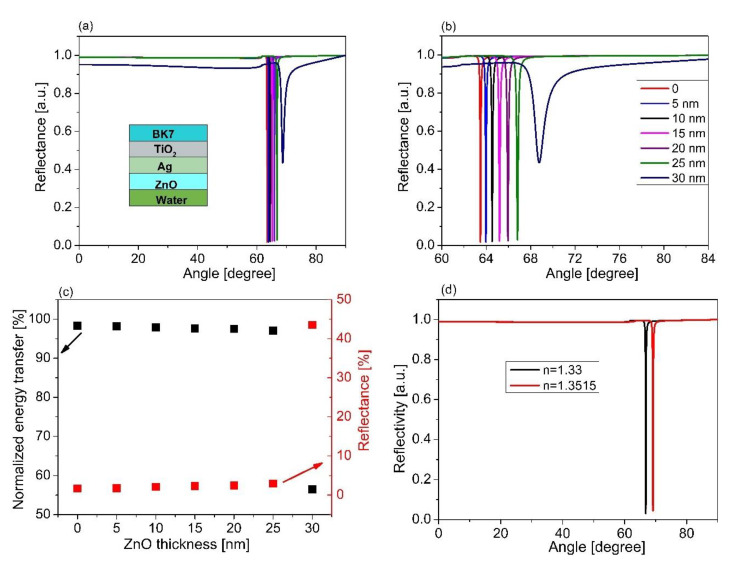
SPR spectra for varying thicknesses of ZnO based on the sensor structure of the BK7/TiO_2_/Ag/ZnO/sensing medium; (**b**) magnified view of (**a**); (**c**) the relationship between energy transfer and the ZnO thickness; (**d**) the change in resonance for different RI of the sensing medium based on the sensor structure of BK7/TiO_2_ (140 nm)/Ag (60 nm)/ZnO (25 nm).

**Figure 8:**
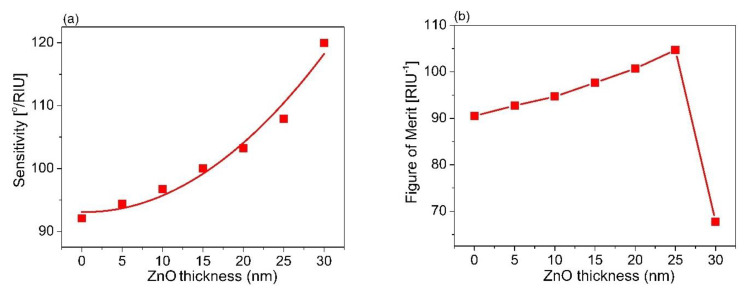
(**a**) The dependence of sensor sensitivity versus the thickness of the ZnO layer; (**b**) The comparison of the FOM of the sensor performance for various ZnO thicknesses.

**Figure 9:**
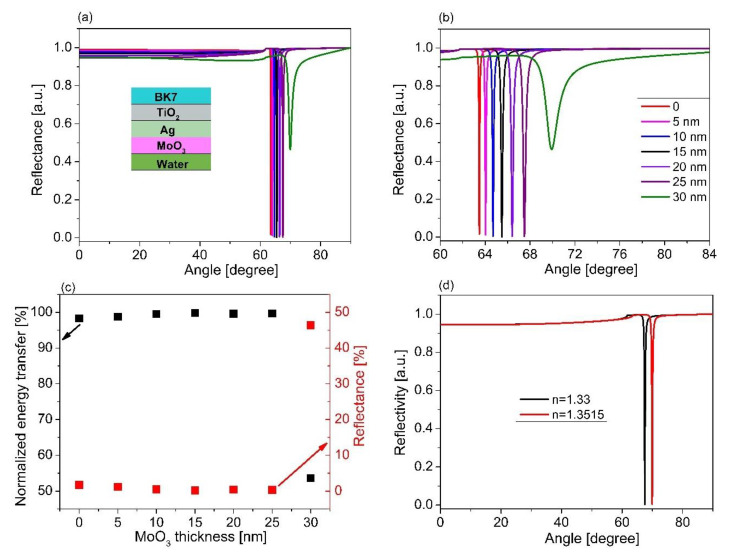
SPR spectra for varying thicknesses of MoO_3_ based on the sensor structure of the BK7/TiO_2_/Ag/MoO_3_/sensing medium; (**b**) magnified view of (**a**); (**c**) the relationship between energy transfer and the MoO_3_ thickness; (**d**) the change in resonance for different RI of the sensing medium based on the sensor structure of BK7/TiO_2_ (140 nm)/Ag (60 nm)/MoO_3_ (25 nm).

**Figure 10:**
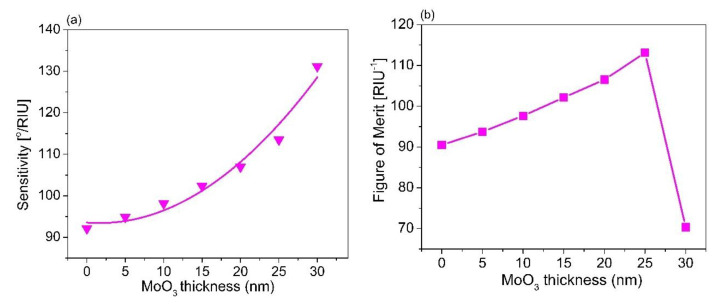
(**a**) The dependence of sensor sensitivity versus the thickness of the MoO_3_ layer; (**b**) The comparison of the FOM of the sensor performance for various MoO_3_ thicknesses.

To check the relevance of the chosen material for SPR sensor performance, AlAs, we also performed control simulations with two more materials, ZnO and MoO_3_, which are usually used as the outermost layer in place of AlAs. Figure 7a represents the SPR spectra for a structure of BK7/TiO_2_/Ag/ZnO with various thicknesses of ZnO. Similar to Figure 5a, SPR dips were observed. It was further obtained that the SPR curve shifted, with an increase in the ZnO thickness. The SPR curve possessed a very low contrast and was quite broad in the case of the ZnO layer thickness of 30 nm (Figure 7b). The energy transfer and reflectivity are also presented as seen in Figure 7c. In addition, the SPR spectra for varying analyte refractive indices based on the optimized configuration of BK7/TiO_2_/Ag/ZnO are shown in Figure 7d. It was observed that the resonant angle increased with an increase in the refractive index of the sensing medium, while the contract of the SPR dip slightly decreased with an increase in the refractive index of the sensing medium. Figure 8a,b present the estimated sensor sensitivity and the FOM for the sensor structure of BK7/TiO_2_/Ag/ZnO, respectively. The results show that the maximum sensitivity and the FOM in the case of BK7/TiO_2_/Ag/ZnO were 1.5 times and 1.45 times lower than that of the case of BK7/TiO_2_/Ag/AlAs, respectively. In addition, Figure 9a illustrates the SPR spectra for different thicknesses of MoO_3_ film in BK7/TiO_2_/Ag/MoO_3_ configuration with the optimum thickness of TiO_2_ (140 nm) and Ag film (60 nm). A similar phenomenon in the case of ZnO film was observed. The resonance angle and contrast, as well as the width of the SPR curves changed with an increase in the MoO_3_ thickness, as shown in Figure 9b,c. In addition, the SPR spectra for varying analyte refractive indices based on the optimized configuration of BK7/TiO_2_/Ag/MoO_3_ are shown in Figure 9d. It was observed that the resonant angle and the contract of the SPR dip increased with an increase in the refractive index of the sensing medium. Moreover, a change in the sensor sensitivity and the FOM were also estimated, which were found to be maximum for the MoO_3_ thickness of 25 nm, as seen in Figure 10. It can also be observed that for the structure of BK7/TiO_2_/Ag/AlAs (as represented in Figure 5d), compared with both BK7/TiO_2_/Ag/ZnO and BK7/TiO_2_/Ag/MoO_3_, the shift in resonant angle was larger for the same refractive index change (Figure 7d and Figure 9d). That led us to obtain better sensitivity for the case of BK7/TiO_2_/Ag/AlAs, as mentioned above.

**Figure 11:**
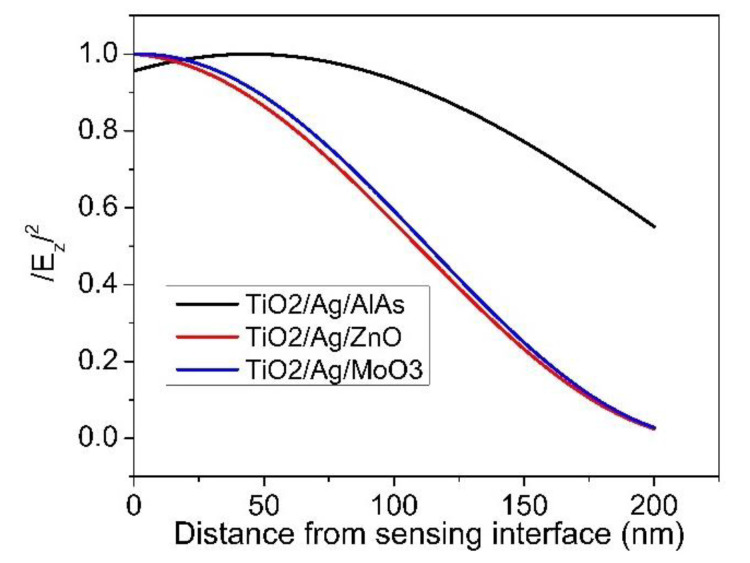
Field profiles for the proposed sensor structures of BK7/TiO_2_ (140 nm)/Ag (60 nm)/AlAs (25 nm); prism/TiO_2_ (140 nm)/Ag (60 nm)/ZnO (25 nm); and prism/TiO_2_ (140 nm)/Ag (60 nm)/MoO_3_ (25 nm).

To demonstrate our point, we plotted the field profiles for three different sensor structures including BK7/TiO_2_ (140 nm)/Ag (60 nm)/AlAs (25 nm); prism/TiO_2_ (140 nm)/Ag (60 nm)/ZnO (25 nm); and prism/TiO_2_ (140 nm)/Ag (60 nm)/MoO_3_ (25 nm), as shown in Figure 11. The simulated results show that the field in the analyte for the sensor structure of BK7/TiO_2_ (140 nm)/Ag (60 nm)/AlAs (25 nm) was stronger than that of the other ones. This was in agreement with the results of the sensitivity analysis and FOM, as discussed above. This phenomenon could be caused by the contribution of the imaginary part of the dielectric constant of the Ag layer, combined with the large real part of the dielectric constant of the AlAs layer, as shown in Table 1. The larger the dielectric constant is, the stronger the field and the deeper the penetration. That also led us to believe that the sensor based on BK7/TiO_2_ (140 nm)/Ag (60 nm)/AlAs (25 nm) can be utilized for the detection of analytes with a larger size, such as bacteria, proteins, and cells. 

The combination of Ag with the other materials such as TiO_2_ and AlAs for implementation of the SPR sensor with the operating wavelength of 1064 nm offers several benefits. The FOM of the SPR sensor based on the combination of TiO_2_/Ag/AlAs is better than that of the sensor using expensive materials such as Au. In addition, the use of TiO_2_ between the prism and the Ag layer provides higher detection accuracy, allowing possible reproducibility of the sensor performance for the detection of bio-targets. Moreover, the simulated results present the possibility of the use of the combination materials with the IR laser for SPR excitation, offering a new research area for bio-sensing applications.

**Table 5:** Performance of SPR-based sensors.

Optical Structure	RI Range	Wavelength	FOM	Reference
TiO_2_/Ag/AlAs-coated prism	1.33–1.3515	1064 nm	~150 RIU^−1^	This work
ZnO-coated U-shaped fiber	1.34–1.42	350–600 nm	~2.4 RIU^−1^	[54]
Au-coated fiber	1.3345–1.3592	300–800 nm	~21.2 RIU^−1^	[55]
Au-coated tapered coreless fiber	1.33–1.391	350–1000 nm	~12.6 RIU^−1^	[56]
Au-coated hetero-core structure fiber	1.333–1385	300–1700 nm	~33.8 RIU^−1^	[57]
S-tapered fiber	1.332–1.387	1200–1600 nm	~125.5 RIU^−1^	[58]
Au-coated fiber	1.30–1.34	300–1700 nm	~61.2 RIU^−1^	[59]
Au-coated few-mode fiber	1.333–1.404	300–1100 nm	~42.2 RIU^−1^	[60]
Au-coated hetero-core structure fiber	1.333–1.3836	400–800 nm	~530 RIU^−1^	[61]
Au-coated fiber	1.335–1.385	360–1700 nm	~13.9 RIU^−1^	[62]
S-tapered fiber	1.336–1.340	1520–1580 nm	~96.1 RIU^−1^	[63]
Ag/Au/MoS_2_-coated optical fiber	1.33–1.37	300–700 nm	~23.29 RIU^−1^	[64]
Au-coated plastic optical fiber	1.34–1.42	360–2500 nm	~25.4 RIU^−1^	[65]

## 4. Conclusions

This work presented the numeric investigation of the SPR sensor with a structure comprising prism/TiO_2_/Ag/AlAs and an operating wavelength of 1064 nm. The resonant spectra can be efficiently realized by adjusting the thicknesses of the TiO_2_, Ag, and AlAs layers. The FOM of the proposed sensor was achieved around 150/RIU, corresponding to the sensor sensitivity of 162.79°/RIU with the optimized thicknesses of the TiO_2_, Ag, and AlAs layers of 140, 60, and 25 nm, respectively. The sensors’ responses were compared with two SPR sensor configurations using ZnO and MoO_3_ in place of AlAs. It is anticipated that the proposed SPR sensor structure can be utilized for various applications such as the quantitative detection of bio-molecules with enhanced figure of merits.

## Figures and Tables

**Table 1:** Parameters of materials used for simulation.

Materials	Wavelength (nm)	Dielectric Constant (ε_r_ + iε_i_)	References
Prism (BK7)	1064	2.28	[33]
Ag	1064	−66.26 + 5.83i	[47]
AlAs	1064	8.64	[48]
TiO_2_	1064	6.15	[49]
ZnO	1064	3.76	[50]
MoO_3_	1064	4.33 + 0.011i	[51]

**Table 2:** Kinetic parameters of energy transfer.

Wavelength (nm)	Minimum Energy Transfer *E_o_* (a.u.)	Fitting Coefficients		
		*α*	*β*	*R* ^2^
1064 nm	163.22	−0.06	7.91	0.89

**Table 3:** Estimation of the sensitivity and detection accuracy of the BK7/TiO_2_/Ag-based sensor.

TiO_2_	80 nm	100 nm	120 nm	140 nm	160 nm
Sensor sensitivity (°/RIU)	92.09	92.09	92.55	92.09	92.55
Detection accuracy (/°)	13.88	13.89	13.89	15.87	15.87

**Table 4:** Kinetic parameters for sensor sensitivity performance with three different materials of outermost layer (AlAs, ZnO, and MoO_3_).

	Sensor Sensitivity *S_o_* (°/RIU)	Fitting Coefficients		
		*a*	*b*	*R* ^2^
AlAs	94.45	−0.61	0.13	0.99
ZnO	93.11	−0.03	0.03	0.96
MoO_3_	93.55	−0.14	0.04	0.96
